# Understanding signaling and metabolic paths using semantified and harmonized information about biological interactions

**DOI:** 10.1371/journal.pone.0263057

**Published:** 2022-04-18

**Authors:** Ryan A. Miller, Martina Kutmon, Anwesha Bohler, Andra Waagmeester, Chris T. Evelo, Egon L. Willighagen

**Affiliations:** 1 Department of Bioinformatics (BiGCaT), NUTRIM, Maastricht University, Maastricht, The Netherlands; 2 Maastricht Centre for Systems Biology (MaCSBio), Maastricht University, Maastricht, The Netherlands; 3 Micellio, Antwerp, Belgium; Texas A&M University College Station, UNITED STATES

## Abstract

To grasp the complexity of biological processes, the biological knowledge is often translated into schematic diagrams of, for example, signalling and metabolic pathways. These pathway diagrams describe relevant connections between biological entities and incorporate domain knowledge in a visual format making it easier for humans to interpret. Still, these diagrams can be represented in machine readable formats, as done in the KEGG, Reactome, and WikiPathways databases. However, while humans are good at interpreting the message of the creators of diagrams, algorithms struggle when the diversity in drawing approaches increases. WikiPathways supports multiple drawing styles which need harmonizing to offer semantically enriched access. Particularly challenging, here, are the interactions between the biological entities that underlie the biological causality. These interactions provide information about the biological process (metabolic conversion, inhibition, etc.), the direction, and the participating entities. Availability of the interactions in a semantic and harmonized format is essential for searching the full network of biological interactions. We here study how the graphically-modelled biological knowledge in diagrams can be semantified and harmonized, and exemplify how the resulting data is used to programmatically answer biological questions. We find that we can translate graphically modelled knowledge to a sufficient degree into a semantic model and discuss some of the current limitations. We then use this to show that reproducible notebooks can be used to explore up- and downstream targets of MECP2 and to analyse the sphingolipid metabolism. Our results demonstrate that most of the graphical biological knowledge from WikiPathways is modelled into the semantic layer with the semantic information intact and connectivity information preserved. Being able to evaluate how biological elements affect each other is useful and allows, for example, the identification of up or downstream targets that will have a similar effect when modified.

## Introduction

Human cells contain around 20,000 protein-coding genes and numerous non-coding genes [1] and each coding gene can encode many proteins. Furthermore, the Human Metabolome Database (HMDB) describes over 100,000 metabolites [2]. The number of interactions between biological entities is even higher. For example, cells also contain many membrane and soluble protein complexes [3], the latter estimated as at least 600 [4], while many more are predicted [5]. The size and complexity of the system gives a system-wide overview, but sometimes breaking the system into smaller pieces that can be used for analysis and experimentation is wanted [6, 7].

WikiPathways is an open source pathway repository that is open to the community to create and modify pathway diagrams so that they can be shared with everyone in the community [8]. The WikiPathways database depicts biological processes and their connections to each other. The connections of elements within a pathway are shown as edges from one node to the next. These edges themselves have biological meaning that can be modelled and represented in WikiPathways [9].

For interoperability, WikiPathways also has a Resource Description Framework (RDF) set associated with it [10]. The RDF is the semantic representation of pathway diagram elements that are displayed and generated from the original Graphical Pathway Markup Language (GPML) in which WikiPathways stores the pathways (see Table 1 for terminology used in this article). The WikiPathways RDF then includes both the graphical RDF (GPMLRDF) and the semantic elements of the RDF (WPRDF). The RDF allows users to go from creating an image of a biological pathway to trapping the elements and keeping them in a machine readable way and made available to be queried. One of the advantages of this is that it is also a linked data resource that can be queried by users at the WikiPathways SPARQL Protocol and RDF Query Language (SPARQL) endpoint, to query RDF databases (http://sparql.wikipathways.org/). This store of the WikiPathways RDF can be accessed both directly from the WikiPathways SPARQL endpoint, but also by remote requests via federated queries.

**Table 1:** Abbreviations for semantic web technologies used to harmonize the biological interaction information from WikiPathways.

Abbreviation	Full Name/Meaning
GPML	Graphical Pathway Markup Language
GPMLRDF	RDF for Graphical Pathway Markup Language
MIM	Molecular Interaction Map
RDF	Resource Description Framework
SBGN	Systems Biology Graphical Notation
ShEx	Shape Expressions
SPARQL	SPARQL Protocol and RDF Query Language
WikiPathways RDF	The combination of GPMLRDF and WPRDF
WPRDF	RDF for WikiPathways

In order to represent connectivity between nodes in a pathway diagram, the meaning of a drawn line connecting nodes needs to be understood. WikiPathways RDF has connectivity information stored as point A is connected to point B. To a human looking at a pathway, it is more obvious what an arrow connecting two points means or what is implied by the arrow, but the RDF needs this stated explicitly if any inferences about how elements are connected is to be gleaned. In fact that is even true when standardised graphical representations for interactions like Molecular Interaction Maps (MIM) [11] and Systems Biology Graphical Notations (SBGN) [12] are used.

Furthermore, to ensure the biological causality is reflected in the graph representation in the RDF, we need to make sure the latter reflects that interactions can be directed and undirected. Information about the direction and connectivity in a pathway diagram helps to explain the biological processes and therefore helps understand cause-effect relationships represented in the pathway. However, not all interactions have a clear direction: while the direction of a metabolic conversion follows chemical thermodynamics, interactions like the associations that exist in a complex are symmetrical and do not have a direction. Even more complex is a ligand binding, where the physical interaction is not only directed, but the interaction arrow also reflects the movement of the ligand. Therefore, it is important to know if an interaction has a directed route as part of a path and the RDF needs to preserve this information.

To ensure that pathway interaction drawings and notations can be biologically interpreted, the RDF needs to have standardized types for the interaction. That will allow users to query for all reactions of a similar (biological) type rather than worry about which notation was used in the drawing. WikiPathways supports several drawing notations, which can be general WikiPathways notations, MIM notations, and SBGN notations. Based upon WikiPathways GPML data model and the underlying ontology, these three can all be used and shown on WikiPathways. The available interactions themselves can be classified into nine different types: conversions, bindings, interactions, directed interactions, catalysis, transcription translation, complex bindings, inhibitions, and stimulations.

**Fig 1:**
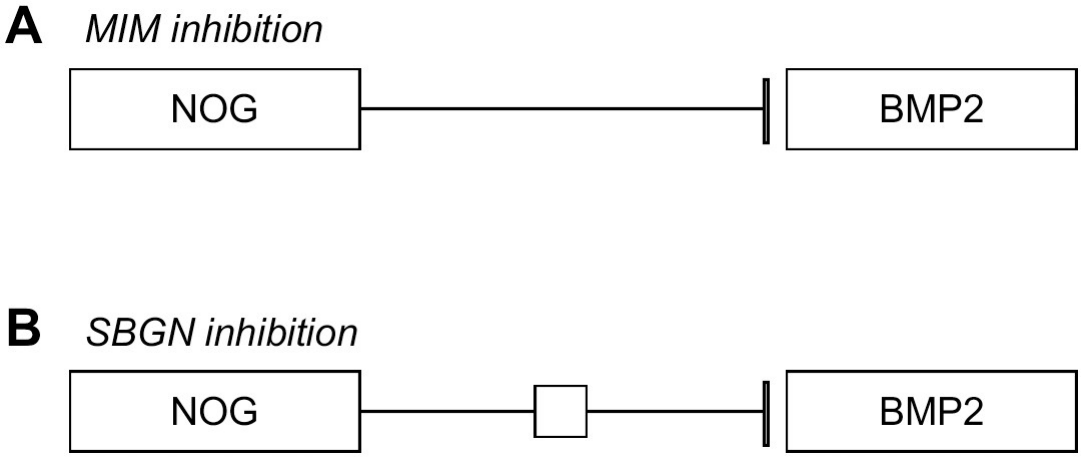
Differences in drawing of MIM vs SBGN inhibition interaction. A shows a MIM—inhibition interaction. B shows a SBGN—inhibition interaction.

When interactions in various notations are normalized, more biological knowledge can be explored, and new questions answered. This interoperability effort makes it possible to gain implied knowledge from how a pathway diagram is drawn. For example, if two enzymes are catalyzing some chemical substrates in succession then there would typically not be a direct link or arrow drawn from one enzyme to the other, but in order for the second enzyme to work the product from the first reaction must be present. This has the implication that the second enzyme is biologically downstream of the first enzyme, even though this interaction is not explicitly drawn. Having semantically clear directions and interaction types is essential to reach this conclusion from the RDF. Drawing of interactions with the WikiPathways and MIM notations can be done with the default installation of the PathVisio core [9], while SBGN needs a PathVisio plugin https://github.com/PathVisio/pathvisio.github.io/blob/master/plugins/sbgn.md. The PathVisio pathway editor thus makes it possible to annotate an interaction as a simple line with an arrowhead, as a MIM interaction, by default, or to create a SBGN drawing using plugins. It then becomes necessary to unify common types from the different graphical standards so that a MIM-Inhibition and a SBGN-Inhibition are understood as the same thing. Fig 1 shows the differences in drawing of an inhibition between SBGN and MIM notation. After all, in both cases, the interaction is indicating an inhibitory effect of one entity upon another. Knowing the interaction types gives important context of the connection and the entities involved. A small note about how complexes are represented is also essential. In the RDF all the entities are connected to each other with an undirected interaction. This keeps them all connected to each other as well as with any interaction that they are associated with as a complex.

The general interaction type is used to denote an interaction between data nodes and thus all interactions are of this type. A directed interaction, on the other hand, means there is a direction that says one data node is influencing another but the exact mechanism is not known, or at least not described by the pathway creator (author). Directed interaction is also the general data type for all interactions that have some directional information included. Therefore, all interactions have the type directed interaction except binding and complex binding, with the directed interaction itself being a child of the general interaction type. We therefore wanted to study to what extent we can derive knowledge from biological interactions, by semantically capturing biological meaning of interactions and harmonizing the notation in pathway drawings. We tested our hypothesis that this can be done by answering the following questions. First, can we translate graphically modelled biological knowledge to a semantic model of biological knowledge that harmonizes interaction types and captures implied directional information And second, can we then take advantage of the semantic translation of the graphical biological knowledge to programmatically answer biological questions. For this latter question, we studied two specific biological questions as examples: in one example we look at MECP2 and explore alternative targets for this protein by looking for targets either upstream or downstream as they both have an effect on MECP2’s role. For the other example we studied how lipid metabolism is captured in the *Ganglio Sphingolipid Metabolism* pathway (wikipathways:WP1423, WikiPathways Project *et al*., 2019).

The description of interaction information allows for the advancement of curation efforts by the WikiPathways team. This curation in turn allows the team to improve the quality of pathways and a more complete overview of which elements are in the pathways and how they are connected to one another. Using SPARQL queries for curation the curators can identify why the interactions are not converted from the graphical description of WikiPathways to the semantic description of the WikiPathways RDF and can explore how to improve this.

When we understand how interactions work we can also pre-define the form or shape that such a specific interaction type takes. For this the Shape Expressions (ShEx) standard can be used [13–15]. A ShEx determines what information is expected for, in this case, a specific interaction type. ShEx will be created for all interaction types in WikiPathways. The shape expression can then be used to monitor translations of knowledge of one format or notation to another, for example, when adding data from one database to another [16]. This allows us to focus more on the biology and less on the bioinformatics, as we get alerted about unexpected shapes.

To explore these approaches, we look at two biological research topics studied in our group: a rare disease and human lipid metabolism. MECP2 is a protein involved in a rare disease and important in the methylation of DNA [17]. Mutations in the MECP2 gene have been linked to the development of Rett Syndrome [18]. This disease is responsible for a host of neurological developmental issues that affects infant development. The MECP2 gene lays on the X-chromosome and Rett Syndrome is found in females [19] because the severity in males is too high for patients to be viable. The severity of the disorder is related to the specific mutation found in the individual patient [20]. Ehrhart *et al*. have already demonstrated the power of integrating different databases to retrieve links between genetic variants and phenotypes [21]. Being able to look at alternative targets that are a part of the sequence of developments that lead to disorders such as Rett may end up helping us to expand the knowledge about alternative causes and treatment opportunities. The types of interactions described for MECP2 are a simple case of connectivity and directional information captured in WikiPathways and make a good example to demonstrate how this can be used to allow observation of upstream and downstream interactions.

The second example describes the metabolic regulation and modifications of sphingolipids which are known to regulate several cell functions [22]. Sphingolipids are produced in the endoplasmic reticulum and the modifications of this lipid class alters the effect of the specific sphingolipid’s function [23]. The conversion of these metabolites from one form to another is regulated by enzymes that act as a catalyst for the reaction to take place. Sphingolipids also play a role in signal transduction [24]. The sphingolipids play an important role in the membrane of eukaryotic cells and are often associated with disorders in the degradation of lipids [25]. This shows the importance of proper metabolite regulation and metabolism as disruptions can lead to serious diseases with high mortality rates. Understanding how these elements of the pathway are connected to one another and how they are directed helps to understand when the elements are not working correctly. There are also a large number of proteins that are known to interact directly with sphingolipids and are necessary for cell function [26]. In WikiPathways, these types of interactions are most often drawn with an arrow that shows the conversion of the metabolites from one form to another along with an associated catalysis reaction that is facilitated by an enzyme. Looking at how metabolism is modelled in wikipathways:WP1423 helps illustrate how these conversion and catalysis reactions are stored. Metabolism interactions are a more complicated set of interactions as an enzyme is typically seen acting on another interaction. The sphingolipid metabolism pathway displays this more complex observation and allows the identification of the order of the enzymes found for potential upstream/downstream analysis.

## Materials and methods

### WikiPathways data

#### Interaction modeling

The interactions in WikiPathways are modeled by taking the graphical semantic information from the pathway diagram’s GPML representation. The harmonization of interactions is part of the WPRDF generation. This is done by analysis of the lines that represent interactions in the graphical representation, and using these to decide how the participants in the interactions are connected. All harmonized interactions have a unique ID, are linked to the participants, and have an interaction type as outlined in the introduction. If it is a directed interaction, it will also have a source and target node for the interaction. JUnit (https://junit.org/) was used to test the harmonization with several tests to verify that these connections in the GPML are being converted to RDF as expected. These tests include the original GPML and the expected outcomes as described in the code repository at https://github.com/BiGCAT-UM/WikiPathwaysInteractions/tree/master/FilesGPML.

#### Benchmark data

We used the RDF from the WikiPathways June 2019 release (https://zenodo.org/record/3369380). Both the WPRDF and the GPMLRDF components of the WikiPathways RDF were used in this study. To examine how pathways are drawn and used in WikiPathways, the analysis used only pathways from the Curated collection and only for *Homo sapiens*, and therefore excludes the Reactome collection [27, 28].

### Data analysis

To aggregate and analyze the date, Jupyter Notebooks running Python were used to collect all SPARQL queries that were used to query the WikiPathways SPARQL endpoint [29]. The notebooks are available from (https://github.com/BiGCAT-UM/WikiPathwaysInteractions/): *DataNodeStats.ipynb*, and *InteractionStats.ipynb*, and two for the two biological examples. The first two represent two different categories of queries. *DataNodeStats* retrieves information about data nodes in both parts of the WPRDF while the *InteractionStats.ipynb* file is used to return data about connectivity between the nodes in the WikiPathways RDF, representing both the semantic and the graphical RDF elements. *ExampleMECP2.ipynb* is the file for the query related specifically to the *MECP2 up and down stream targets* example. Finally, *ExampleLipidMetabolism.ipynb* is the notebook for the case of *sphingolipid metabolism*. These notebooks and their use are further described below.

#### Datanode harmonization

Data nodes needed to be harmonized first in order to be able to examine the connections between the nodes. There are two conditions that determine the conversion of the interactions: the participating datanodes are converted, and second, the interaction is converted. That allowed us to better estimate how well the interaction harmonization itself went. Therefore, we first looked at the data nodes. The *DataNodeStats.ipynb* notebook contains Python code to calculate a series of counts of data nodes, to estimate the amount of data and to get a baseline number of what we can expect for the success of conversion and harmonization of interactions. It is important to realize that for interactions where one of the participating data nodes is not in the WPRDF, the conversion script will not to be able to create the interaction due to the absence of participants. Therefore this interaction will not be found in the WPRDF and will affect our interaction counting. The notebook calculates the total number of data nodes of a certain type, in the Jupyter Notebook section *Datanode Type Counts*, and the corresponding numbers of GPMLRDF data nodes without a WPRDF data node equivalent. Furthermore, it determines the number of GPMLRDF data nodes of type complex without WPRDF equivalents. This is used to specifically track which data nodes that are part of the complexes that can be found in the graphical elements part of the RDF but not found in the WPRDF, the biological component of the WikiPathways RDF. These complexes are not annotated as biologically known complexes. Those exist because the biological meaning of complexes is currently not always well-defined in pathway drawings in WikiPathways.

#### Interaction harmonization

The *InteractionStats.ipynb* notebook contains code to calculate numbers that reflect the harmonization of interactions in the biological WPRDF, by taking into account the different drawing notations as a unified interaction type. The first few sections calculate overall statistics, the *Number of Non-Directed Interactions* (for example, bi-directional binding), *Count of Interaction Types* (reflecting the biological nature of the interaction), *Interaction Count with Unspecified Type*, and the percentage of non-directed interactions. The second set of sections characterize the nature of the interactions, e.g. *Interaction counts by participants*, *Participants for Interactions* (which reflects what datanode types are involved in an interaction), and *Identifier IDs by data source*.

In order to evaluate the conversion success, it calculates the complementary *GPMLRDF Interactions without a WPRDF equivalent* and *GPMLRDF Interactions with a WPRDF equivalent*, and the resulting percentages of success (see *GPMLRDF Interaction with Equivalent WPRDF out of Total GPMLRDF Interactions*). The GPMLRDF Interactions without a WPRDF equivalent was used to check to see how many interactions that are present in the graphical version of the RDF but not present in the biological WPRDF. The query for the percentage of WPRDF Interactions that are of unspecified type was used to see how accurately detailed the biological pathways are annotated. Finally, the percentage of non-directed interactions in the notebook calculated how many of the WikiPathways interactions are of non-directed type. When these are between metabolites and they may reflect missing biological annotation of directions.

### Usability

To test our hypothesis that we can harmonize the interaction information, we developed the Jupyter Notebooks to first collect and query the data from the WikiPathways RDF. We then created several unit tests to validate how the modelled interactions behaved and to verify that they are created correctly. This ensures that when an interaction is drawn, we can keep track of the relevant semantic data represented, such as what nodes are connected to each other, what type of interaction is drawn between them, and how many nodes are expected to be part of the interaction. We can then test assumptions like: “interactions between metabolites should be directed conversions” and “interactions between different proteins should not be conversions” and add other aberrant results as curation tasks. We further tested with two biological examples if the harmonized semantified interactions give interpretable answers.

#### Curation

The Jupyter Notebook created for interaction curation uses the query for GPML RDF interactions without a WP RDF equivalent to generate a list of interactions that are not found in the semantic portion of the RDF. The next query in the notebook finds the specific elements for the interactions in this list that will help the curator identify which elements are missing. The query includes the interaction ID for the GPML RDF, the pathway in which it can be found, and the connecting elements found on either end of the interacting line.

#### ShEx

Shape expressions were created manually for the modelled WikiPathways interactions. ShEx for WikiPathways interactions were formed following the standards laid out by the ShEx project (https://shex.io/). These shape expressions can be found in the shape expressions subdirectory on the GitHub repository (https://github.com/BiGCAT-UM/WikiPathwaysInteractions/tree/master/ShExInteractions). The harmonized interaction types were expressed as ShEx. ShEx can be used for curation events to verify that the interaction fits the shape that is expected by the WikiPathways model, and in this way help detect data issues. The npm module shex (https://www.npmjs.com/package/shex) was used to run the shape expression on the harmonized model. A GNU/Linux Makefile on GitHub demonstrates the combination of SPARQL to list all resource IRIs of a certain interaction type and the JSON query tool jq (https://stedolan.github.io/jq/) to process the ShEx module output to count the number of errors for each interaction. This allowed running the shape expression on all directed interaction in the WPRDF.

#### MECP2 up- and downstream targets

For the specific example used for MECP2 metabolism, the Jupyter Notebook used a SPARQL query to the WPRDF. This query works by first searching for targets that are upstream or downstream of MECP2. The query then identifies data nodes that are associated with the HGNC symbol MECP2. The query in the Jupyter Notebook finally finds associated pathways that have this HGNC symbol present and matches interactions that have MECP2 as a target in the interaction.

#### Sphingolipid metabolism

In the case of the specific example used for sphingolipid metabolism, the Jupyter Notebook used a SPARQL query to the WPRDF. The query retrieves the source portion of an interaction and displays its label. In the case of sphingolipid metabolism, the queries identified enzymes that are associated with conversions in the pathway and returned results with the enzyme, interaction, the source metabolite and the target metabolite product.

## Results

To understand the amount of data that can be accessed via the RDF, we looked at the available RDF data for WikiPathways as GPMLRDF and WPRDF, the first being a direct translation of the original graphical depiction of the GPML files and the second covering the biological content. A quick count of the June 2019 release shows that the WPRDF used in this paper had 24,220 data nodes, and 13,928 interactions and is available at http://data.wikipathways.org. The subject of the paper is the interactions between data nodes, but we first need to understand that edges of a network connect datanodes to one another and so understanding the fundamentals of the biomolecular data nodes is necessary. This defines some context for the following results.

### Datanode results

With regards to the data nodes, because of the hierarchical annotation the most prevalent node type is the general datanode type. It is the base type for any datanode, as described by the WikiPathways Vocabularies (https://vocabularies.wikipathways.org) and thus is used for every data node, it may include any of the descriptive data types. More specific but still generic, the GeneProduct type is the next most prevalent node type. These include explicitly typed proteins and RNAs and while the remaining GeneProduct typed nodes are not specified further. Table 2 illustrates the size of the WikiPathways semantic RDF part and the types of nodes present in WikiPathways. There are a total of 28,402 data nodes, the majority of which are gene products. Proteins are the next common type followed by metabolites and RNA. There are also Complex nodes to represent clustered groups of other node types, specifically proteins, gene products, and RNA. Pathways are not typed as Datanode in the WPRDF, which is why the value is blank in the table. Overall, 7.0% of GPMLRDF data nodes do not have a WPRDF data node equivalent and thus 93.0% of the GPML data nodes are found in both parts of the RDF.

**Table 2:** Datanode type counts, as defined by the WikiPathways ontology. The datanode counts for each type of node found in the WikiPathways RDF.

Datanode Type	Count (WPRDF)	Count (GPMLRDF but not WPRDF)
Datanode	28402	—–
GeneProduct	21270	1084
Protein	8255	141
Metabolite	4038	219
RNA	1204	66
Complex	980	16
Unknown	—–	218
Pathway	—–	250

Also seen in Table 2 are the data nodes that are found in the GPMLRDF but not found in the WPRDF. The reason typically is that the node exists but is not linked to a clear biomolecular database identifier, in other words we do not know exactly what it is. Datanodes are any node type in the pathway diagram and the count of gene products also includes proteins and RNAs as these are specifications of the products produced. Complexes are a combination of several other node types that form a unit with one another. We can also see how many data nodes are found in both parts of the RDF.

If we specifically look at some examples of data nodes that are present in the GPMLRDF but not carried over to WPRDF, we can see a list of sixteen complex data nodes, and the details of these are given in S5 File. This second table also includes the labels for the complexes, shedding some light on which complexes were not transferred over to the semantic portion (WPRDF) of the RDF from the graphical portion (GPMLRDF). For all these nodes, they lacked database identifiers.

When we do this evaluation for the pathways of the two use cases, we find that for wikipathways:WP4312, which pertains to MECP2, there is 1 gene product type data node that is found in the GPMLRDF but not found in the WPRDF. This represents 1 gene product out of 148 other gene products that were found in the WPRDF and out of 152 total data nodes found in the WPRDF. In the instance for wikipathways:WP1423, which is related to sphingolipid metabolism, there is 1 metabolite that is found in the GPMLRDF but is not found in the WPRDF. This is 1 metabolite from 38 total metabolites found in the sphingolipid metabolism pathway and out of 62 data nodes found in the WPRDF for wikipathways:WP1423. The last metabolite (Gal-GlcNAc-GM1b) is modified with two sugars, and not found in the reference databases. Future WikiPathways releases can annotate such nodes with the InChIKey, for which no database record is required.

In the S1 File there are tables with examples of data node types that are found in the GPMLRDF but not in the WPRDF for various pathways (as counted in Table 2). In this file, the query results are retrieved along with the table to give some idea why they may not be translated. In S2 and S3 Files there are tables for the data node counts for the specific WikiPathways example pathways of MECP2 and sphingolipid metabolism.

### Interaction results

Similar to what we did for the data nodes, we calculated non-directed interactions and non-specific interactions along with the specific interaction types and counts. Non-directed interactions being all interactions that do not have any directional information, such as in the case of a binding event. Non-specific, on the other hand, means that an interaction does not even have a specified non-directed interaction like a binding.

**Fig 2:**
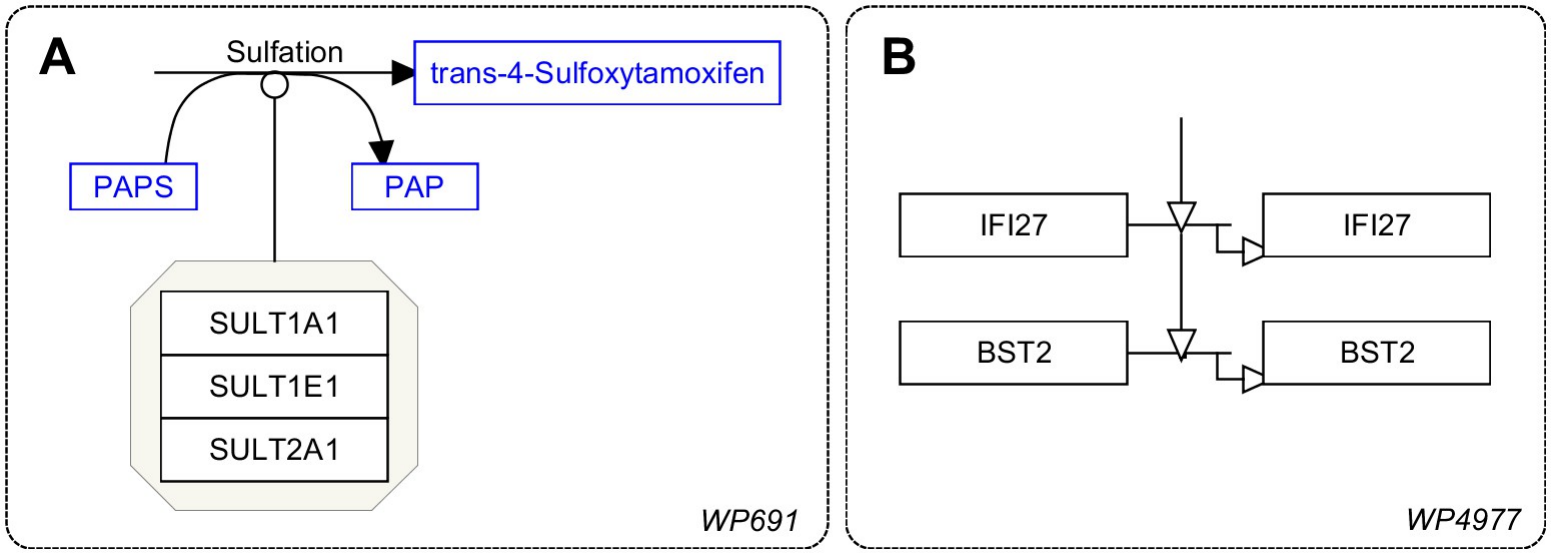
Interaction types that are not found in Table 6. A shows a complex binding of SULT1A1, SULT1E1 and SULT2A1 that catalyzes cis-4-hydroxytamoxafin to trans-4-sulfoxytamoxifen with PAPS to PAP formation found in Tamoxifen Metabolism (wikipathways:WP691). B shows transcription translation interaction for BST2 to BST2 in Host-pathogen interaction of human corona viruses—MAPK signaling pathway (wikipathways:WP4877).

First, we identified nine interaction types. The overview of mappings to WPRDF of the GPML interaction types that can be found in WikiPathways, is available from https://github.com/BiGCaT-UM/WikiPathwaysInteractions/tree/master/FilesGPML. The nine types of interactions found in the GitHub page are catalysis, complex binding, conversion, general undirected interaction, inhibition, stimulation, transcription/translation, an unspecified directed interaction, and a directed interaction with multiple inputs and multiple outputs. This GitHub repository contains example GPML files for each interaction type that can be found at https://vocabularies.wikipathways.org/, along with an example of what the interactions look like in GPML, as well as files with statistics about the interaction as it appears in the WPRDF. These numbers are used in the JUnit tests to verify that the different models are harmonized into the single interaction model in WPRDF. These tests are now available as part of the regular testing of RDF generation (see https://github.com/wikipathways/GPML2RDF, *src/test/java/org/wikipathways/wp2rdf/interactionTests* folder). When we look at the full WPRDF, the types of generic non-directed and nonspecific interactions can be seen. Out of a total of 15,525 interactions, 3,706 (23.9%) were non-directed of which 2,766 (17.8%) were non-specific (see Table 3). Thus 11,819 (58.3%) of the interactions have some sort of direction information. The number of non-specific interactions can be either an indication that there is just not sufficient evidence to explain what the interactions are or that better curation is necessary. Examples of how interactions are drawn in WikiPathways can be seen in Fig 2.

**Table 3:** Interaction type counts, as defined in the WikiPathways ontology. The sum of DirectedInteration and NonDirected equals the Interaction Total. Of the directed interactions, subsets are typed as Conversion, Inhibition, etc. The NonSpecified interactions is a subset of NonDirected interactions. More than 12 thousand interactions are only found in the GPMLRDF.

Interaction Type	Count (WPRDF)	Count (GPMLRDF but not WPRDF)
Interaction	15525	—–
DirectedInteraction	11819	—–
Conversion	1447	—–
Inhibition	1091	—–
Catalysis	1231	—–
ComplexBinding	940	—–
Binding	1513	—–
Stimulation	842	—–
TranscriptionTranslation	256	—–
NonDirected	3706	—–
NonSpecified	2766	—–
Unknown	—–	12287

Only a small percentage of the interactions have associated identifiers. Having such identifiers can make it easier to find information about the provenance of that interaction occurring in a pathway and it is useful for linking experimental data or modelling results to the pathway or to find descriptions of the interactions in external resources. Table 4 contains provenance information about the databases to which identifiers for interactions refer. UniProt-TrEMBL has the most interactions represented in WikiPathways. There were some unexpected database links. Sources like *kegg.compound* and *ChEBI* are not expected to have interaction data information but are included because the user identified them as the database resource for the interaction. These unexpected sources come from two pathways, wikipathways:WP3634, and wikipathways:WP3635. These two pathways use very specific notation and while unexpected, have been intentionally annotated like this. These pathways use the SBML notation and represent the normal versus disease state of insulin signaling [30]. Generally, the main reason that currently most interactions do not have any database identifier associated with them is that the mechanism to add these is relatively new.

**Table 4:** Interaction Identifier ID counts by data source. The identifier types for the interactions with a source from the WikiPathways RDF.

Database Source	Interactions
Rhea	313
Uniprot-TrEMBL	213
KEGG Pathway	28
pato	8
kegg.compound	8
ChEBI	6
KEGG Reaction	3
Reactome	3
WikiPathways	2
XMetDB	2
SPIKE	2
BIND	1

Finally, to further characterize the interactions present, Tables 5 and 6 provide examples of the makeup of the interactions seen in WikiPathways. Table 5 shows example Interaction IDs, along with their interaction types, and what type of datanode type is participating in the interaction. And Table 6 shows the profile with the interaction participants and a count of how many times this interaction profile was counted in WikiPathways and the type of these interactions.

**Table 5:** Participants for interactions. Twenty example interaction syntaxes shown in table below. First twenty interactions from the WikiPathways RDF along with their interaction type and the participants for each interaction.

Interaction	Interaction Type	Interaction Participants
WP3668_r97639/ComplexBinding/b916e	Binding	Complex, GeneProduct
WP2879_r94789/ComplexBinding/c939e	Binding	Complex, GeneProduct, Metabolite
WP4262_r97132/ComplexBinding/dae4b	Binding	Complex, GeneProduct, Metabolite
WP585_r94686/WP/Interaction/ida141949	Catalysis	GeneProduct, Protein
WP2533_r95594/WP/Interaction/adbe3	Catalysis	Conversion, DirectedInteraction, Interaction, Protein
WP1601_r95004/WP/Interaction/ida833b0dc	Catalysis	Conversion, DirectedInteraction, GeneProduct, Interaction
WP1423_r94289/WP/Interaction/idde73da53	Catalysis	DirectedInteraction, GeneProduct, Interaction
WP3865_r88186/ComplexBinding/d5e4f	ComplexBinding	Complex, GeneProduct
WP2446_r87639/ComplexBinding/e75ff	ComplexBinding	Complex, GeneProduct, Protein, Rna
WP2795_r97631/ComplexBinding/b5fa4	ComplexBinding	Complex, GeneProduct, Protein
WP3580_r96434/WP/Interaction/id6d378f23	Conversion	Metabolite
WP134_r94935/WP/Interaction/a5dec	Conversion	Metabolite
WP3627_r90137/WP/Interaction/id14d637fe	Conversion	Metabolite
WP2436_r97673/WP/Interaction/b1b2f	Conversion	Metabolite
WP4149_r94399/WP/Interaction/id30000f59	Inhibition	GeneProduct, Protein
WP2261_r89520/WP/Interaction/id65877034	Inhibition	GeneProduct, Protein
WP306_r97459/WP/Interaction/e8847	Inhibition	GeneProduct, Protein
WP2526_r96312/WP/Interaction/ddfe1	Stimulation	Protein
WP1984_r95143/WP/Interaction/id8ba5f251	Stimulation	GeneProduct, Metabolite
WP1984_r95143/WP/Interaction/iddde89331	Stimulation	GeneProduct, Protein

**Table 6:** Top 20 most occurring directional interactions by participants combination. The most abundant interaction is a directed interaction between two metabolites. Interaction participants, the count of how many there are in the WikiPathways RDF and the type of interactions are shown.

Interaction Participants	Count	Type
Metabolite, Metabolite	2675	DirectedInteraction
GeneProduct, GeneProduct	1423	DirectedInteraction
GeneProduct, Protein, GeneProduct, Protein	1334	DirectedInteraction
Metabolite, Metabolite	1125	Conversion
Metabolite	474	DirectedInteraction
GeneProduct, Protein, GeneProduct	445	DirectedInteraction
GeneProduct, GeneProduct, Protein	438	DirectedInteraction
GeneProduct, Protein	420	DirectedInteraction
GeneProduct	315	DirectedInteraction
DirectedInteraction, Interaction, GeneProduct	315	DirectedInteraction
GeneProduct, Protein, Protein	292	DirectedInteraction
Metabolite, GeneProduct	291	DirectedInteraction
DirectedInteraction, Interaction, GeneProduct	274	Catalysis
Protein, Protein	273	Stimulation
GeneProduct, GeneProduct	270	Inhibition
Protein, Protein	262	DirectedInteraction
DirectedInteraction, Interaction, Conversion, Protein	227	DirectedInteraction
DirectedInteraction, Interaction, Conversion, Protein	226	Catalysis
GeneProduct, Metabolite	180	DirectedInteraction
GeneProduct, DirectedInteraction, Interaction	151	DirectedInteraction

When the *PathwayStatsMECP2.ipynb* and *PathwayStatsSphingolipid.ipynb* notebooks were applied to the pathways of the two use cases, we found that for wikipathways:WP4312, which pertains to MECP2, there are 5 interactions that are found in the graphical GPMLRDF but not found in the semantic WPRDF. This represents 5 interactions out of 45 non-specified interactions that were found in the WPRDF and out of 37 directed interactions found in the WPRDF. In the instance for wikipathways:WP1423, which is related to sphingolipid metabolism, there are 24 interactions that are found in the GPMLRDF but not found in the WPRDF. Still, we find 49 directed interactions in the WPRDF for the sphingolipid metabolism pathway, of which 13 are typed as catalytic reactions.

In the S2 and S3 Files tables can be found for the interaction counts of the two specific pathways for MECP2 and sphingolipid metabolism. These contain the types of interactions found in these pathways as well as how many interactions were found in the GPMLRDF but not in the WPRDF resources for WikiPathways as described above.

### Curation

As can be seen in the Jupyter Notebooks for curation, 11081 interactions are found in the GPMLRDF but not found in the WPRDF. The details for the first 20 results are found in Table 7. As can be seen in the table, the query identifies the interaction information from the GPMLRDF, the graph reference ID from the GPMLRDF, and the label for the participants. This can be used to help identify problematic interactions that are not being converted to the WPRDF.

**Table 7:** Curation query showing interaction, GPML graph ref from the WikiPathways RDF, and label for node at end of interaction.

GPML Interaction	GPML Graph Ref	Participant Label
WP107_r105846/Interaction/d2818	e82	EIF4E
WP107_r105846/Interaction/cc170	ceb	ITGB4BP
WP107_r105846/Interaction/f3bb6	fc8	EIF5A
WP1403_r106688/Interaction/ide379f87c	b9666	GLUT4
WP1403_r106688/Interaction/b1235	f344c	Calcium
WP1403_r106688/Interaction/c4810	c9726	FA Synthase
WP1403_r106688/Interaction/f8d22	d9cf5	cAMP
WP1403_r106688/Interaction/d8a35	a84ee	Leptin
WP1403_r106688/Interaction/b166c	ad4a4	Malonyl-CoA
WP1403_r106688/Interaction/e0f9b	d4875	Fatty Acid Oxidation
WP1403_r106688/Interaction/af18d	dcd84	MEF2B
WP1403_r106688/Interaction/e4288	b35fe	Torc2
WP1403_r106688/Interaction/c0527	aeb8f	HMG CoA Reductase
WP1403_r106688/Interaction/cff59	d8c91	HuR
WP1403_r106688/Interaction/ae70c	b3840	Metformin
WP1403_r106688/Interaction/d14e4	b2489	Glucose
WP1403_r106688/Interaction/bedc0	af2e8	Raptor
WP1403_r106688/Interaction/c7163	f156e	PI3K (III)
WP1403_r106688/Interaction/a04e2	df1d0	HNF4A
WP1403_r106688/Interaction/d7df8	f3d7e	4E-BP1

### ShEx

**Fig 3:**
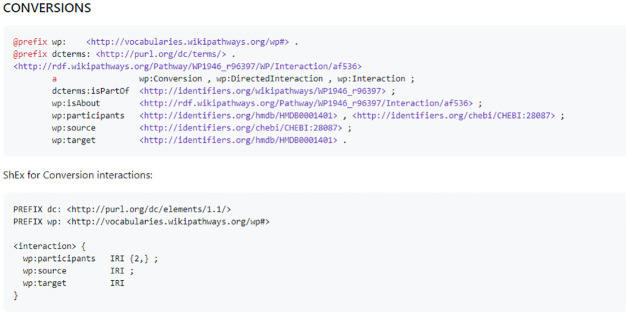
Example ShEx shape for the WikiPathways harmonized Conversion interaction element (RDF shown in the top half), that requires two or more participant IRIs and exactly one source IRI and one target IRI.

All of the ShEx forms can be found on the GitHub repository https://github.com/BiGCAT-UM/WikiPathwaysInteractions/tree/master/ShExInteractions. The interaction types found at https://vocabularies.wikipathways.org/ are general WikiPathways interactions (wp:Interaction), the general WikiPathways directed interactions (wp:DirectedInteraction), the harmonized WikiPathways binding (wp:Binding), complex binding (wp:ComplexBinding), coversions (wp:Conversion), inhibitions (wp:Inhibition), catalysis (wp:Catalysis), stimulations (wp:Stimulation), and transcription-translation(wp:TranscriptionTranslation) interactions. For example, the shape expression representation for a conversion interaction is seen in Fig 3. These represent the harmonized interaction types found in the WikiPathways RDF and their expression in ShEx.

### MECP2 up and down stream targets

**Fig 4:**
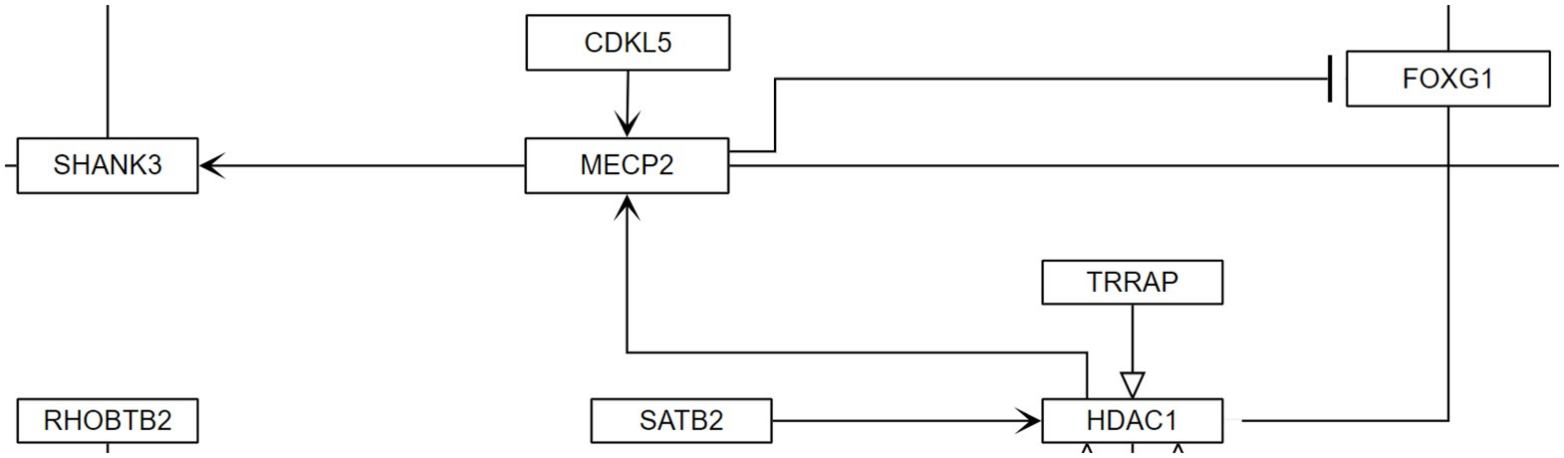
Example of direct interactions of gene products that both influence MECP2 and are influenced by MECP2 from Rett syndrome causing genes (wikipathways:WP4312). In this example, MECP2 is being influenced by HDAC1 and CDKL5. MECP2 then in turns influences SHANK3 and inhibits the activity of FOXG1.

We created Jupyter Notebooks to evaluate the example pathways, as described in the Methods section. The SPARQL queries used in the Jupyter Notebooks will return the interactions that have MECP2 as a participant and then the associated upstream source of the interaction or the associated downstream target of MECP2 and can be found in Table 8. Fig 4 shows examples of the directed nature of influences by MECP2. The query identified ten gene products that are known to influence or be influenced by MECP2. Three gene products were upstream of MECP2 and have an influence on MECP2, while the other 7 gene products were downstream of MECP2 and indicate that they are influenced by MECP2. This basically captures the semantics of the biological meaning of the pathway, a rare disease caused by a damaged gene that has a variety of effects and interactions.

**Table 8:** MECP2 upstream and downstream targets. In the table a source node is shown with its label, as well as the target and its label. The pathway in which the interaction is found and the interaction id are also provided.

Source	Source Label	Target	Target Label	Pathway	Interaction
ensembl:ENSG00000169057	MECP2	chebi:CHEBI:29987	glutamate	wikipathways:WP3584_r96364	Interaction/id4f207df3
ensembl:ENSG00000169057	MECP2	chebi:CHEBI:29987	Glutamate	wikipathways:WP3584_r96364	Interaction/id4f207df3
ensembl:ENSG00000169057	MECP2	ensembl:ENSG00000118260	CREB1	wikipathways:WP3584_r96364	Interaction/ida4a8b443
ensembl:ENSG00000169057	MECP2	ensembl:ENSG00000118260	CREB	wikipathways:WP3584_r96364	Interaction/ida4a8b443
ensembl:ENSG00000169057	MECP2	ensembl:ENSG00000176697	BDNF	wikipathways:WP3584_r96364	Interaction/id4a259c62
ensembl:ENSG00000169057	MECP2	ensembl:ENSG00000155511	GRIA1	wikipathways:WP3584_r96364	Interaction/id3bcd32
ensembl:ENSG00000169057	MECP2	ensembl:ENSG00000155511	AMPA	wikipathways:WP3584_r96364	Interaction/id3bcd32
ensembl:ENSG00000169813	HNRNPF	ensembl:ENSG00000169057	MECP2	wikipathways:WP3584_r96364	Interaction/id1c3def3d
ensembl:ENSG00000196132	MYT1	ensembl:ENSG00000169057	MECP2	wikipathways:WP3584_r96364	Interaction/id8e7af5c
ensembl:ENSG00000169045	HNRNPH1	ensembl:ENSG00000169057	MECP2	wikipathways:WP3584_r96364	Interaction/ida6a9fa9d

### Sphingolipid metabolism

**Fig 5:**
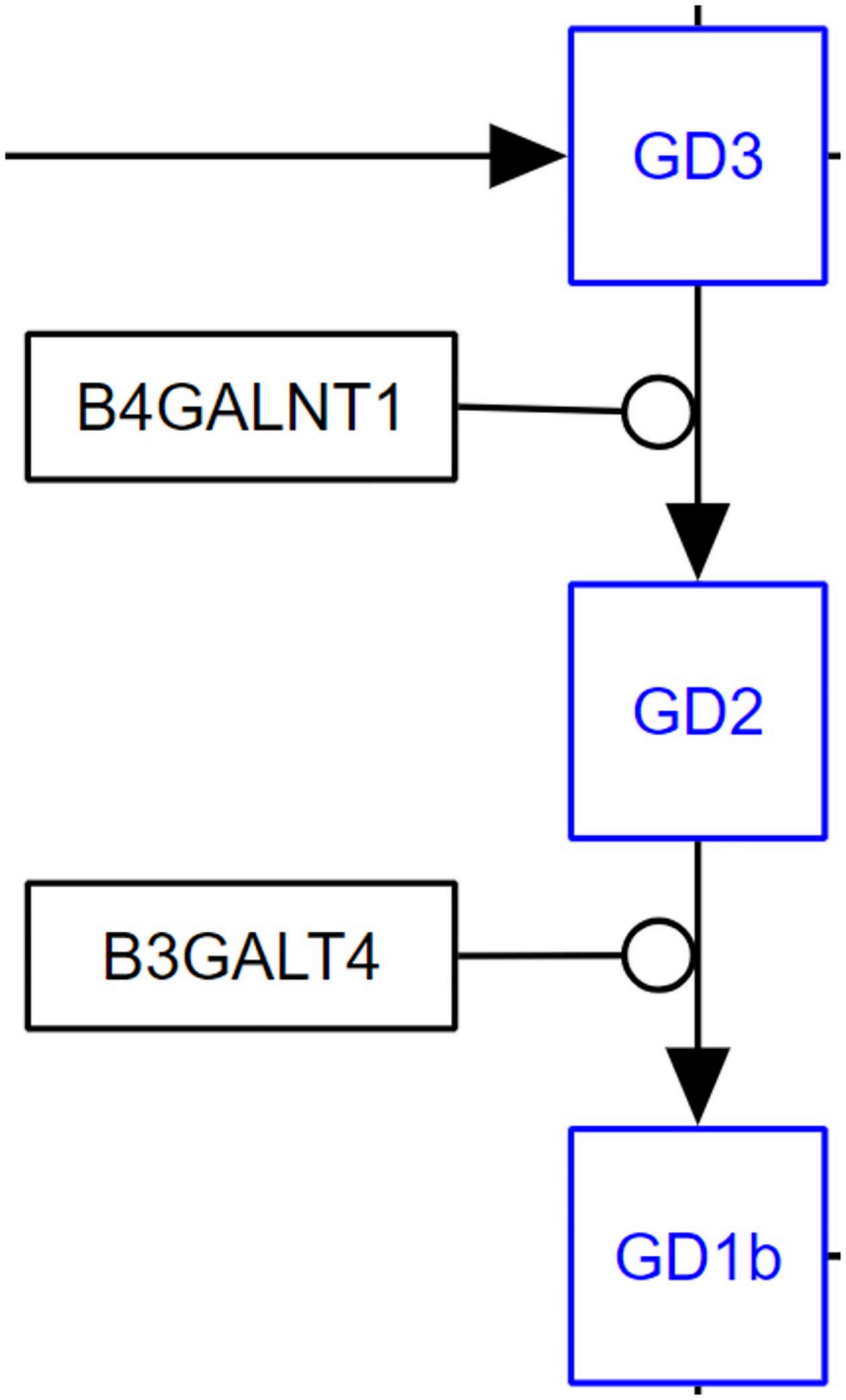
Representation of conversion of different sphingholipids to their products and the relevant enzyme catalyzing the reaction from the Ganglio Sphingolipid Metabolism pathways (wikipathways:WP1423). In this case, GD3 is converted to GD2 by the enzyme B4GALNT1. GD2 is then in turn converted to GD1b and catalyzed by B3GALT4.

For sphingolipid metabolism, a Python script was devised that queries the WPRDF for WikiPathways pathway wikipathways:WP1423, Ganglio Sphingolipid Metabolism, and returns a table with directed interactions that have an enzyme that is catalyzing the reaction. The query limits results to wikipathways:WP1423 as a matching criteria, then finds interactions that are annotated as being a catalysis reaction. It retrieves the associated protein for the catalysis along with the interaction that is being acted upon. Finally, the query also retrieves the source (substrate) and target (product) for the directed interaction that was being catalyzed. Fig 5 shows an example enzymatic reason. The results of the query are shown in Table 9, five conversion annotated interactions in this pathway were returned.

**Table 9:** Sphingolipid conversion interactions. In the table the enzyme for the conversion is given along with the metabolite source and its label along with the metabolite target along with its label and completed with the interaction id for the conversion.

Enzyme	Metabolite Source	Source Label	Metabolite Target	Metabolite Target Label	Interaction
ensembl:ENSG00000115525	hmdb:HMDB0006750	Lactosylceramide	hmdb:HMDB0004844	GM3	Interaction/idb121743e
ensembl:ENSG00000115525	hmdb:HMDB0006750	LacCer	hmdb:HMDB0004844	GM3	Interaction/idb121743e
ensembl:ENSG00000169359	hmdb:HMDB0004913	GD3	pubchem.compound:73427362	O-Acetylated GD3	Interaction/id5f3f21f
ensembl:ENSG00000235863	hmdb:HMDB0004925	GD2	hmdb:HMDB0004926	GD1b	Interaction/idde73da53
ensembl:ENSG00000101638	hmdb:HMDB0004927	GT1b	hmdb:HMDB0004928	GQ1bA	Interaction/idc09b2721

## Discussion

The analysis in this paper only involves human pathways on WikiPathways from the original, non-Reactome, collection. For other species, the results would have been affected by the more limited curation effort that has been spent on those in general. To allow us to do meaningful interaction analysis we need to have sufficient information about the interactions and their participants. Generally, a data node might be found in the graphical portion of the RDF and not in the semantic portion because of incorrect annotations, because the curator really meant to add something atypical, like an organ, or because of a failure by the conversion scripts to successfully convert the graphical information into semantic information.

Interaction types were harmonized by the scripts to turn pathway graphical information into semantic data if there was an appropriate analogue and drawing for the different notation types. This allows for example, a user to draw either a SBGN, a MIM, or a general WikiPathways inhibition drawing to have a harmonized interaction type called wp:Inhibition. In this example, since all three different notation types have the same biological meaning of indicating an inhibition event, it allows the user the flexibility to draw the pathway in the notation they are most comfortable using and still preserving the meaning of the interaction edge.

In addition to harmonization of the WikiPathways interaction types, it is shown to be possible to represent the interactions as shape expressions or ShEx. ShEx were created for all the harmonized interaction types that are found in WikiPathways. The ShEx for an interaction informs the user what is expected to be found from a certain resource. For interactions, this means it is possible to know the general shape to expect for any interaction found within the WP RDF.

For the more curated human pathways, we find that gene products that are in the GPMLRDF but not in the WPRDF, typically these are nodes that do not have a selected database resource type, like Ensembl or NCBI Gene. From Table 2 we learn that most of the data nodes already do have enough information to be included in the semantic part of the RDF. Future curation tasks to identify appropriate sources for the data nodes with missing annotations would enable them to become part of the semantic information. Curation efforts are a part of improving the quality of WikiPathways as a resource but also improving the coverage of interacting elements that are queryable by biologists that are looking to explore their genes or processes of interest.

Three further examples of existing problems with data nodes exist for nodes of unknown type, pathway nodes, and complex data nodes. The unknown nodes do not have an associated data type or an associated database. Pathways nodes are currently part of the WPRDF data model, but only typed as data node and not as pathway, and therefore only get counted as data node.

In the case of data nodes for complexes, there were only 18 complex nodes that do not have an equivalent in the semantic information. These complex data nodes also share the problem of missing database resource or missing data node identifiers, and therefore cannot be converted into WPRDF.

We also saw how data node types and interaction types complement each other. For example, Table 4 shows specific interactions as well as the type of the interaction and the interaction’s participants. This can also be a useful aid in helping to identify areas of curation that need to be addressed. For example, if the participants retrieved for a conversion reaction are metabolites then this makes sense, but if the participants are proteins then there is a possibility that a post-translational modification is described but it is also possible that the user used the wrong annotation for the interaction type, especially when the two proteins are known to be derived from different genes. Based upon the results summarized in Table 5, we can get an estimate of what combinations of participant and interactions types are most prevalent. This gives us an indication of the accuracy of the data. For example, we found a large number of directed interactions connect two metabolites without a specific type. These are likely conversions but they still miss that typing.

We further found that one reason why interactions are captured by the GPMLRDF but not the WPRDF is because some interactions are lines connecting one or more text labels. These are not converted into the semantic layer. The WikiPathways database also allows information added as graphical annotation for the user to better understand a pathway diagram and to provide background information. This type of graphical annotation is only visually curated data but is not meant to show up in the WPRDF.

A third reason why some interactions are not captured in the semantic layer is because one of participants is a user defined group or complex. Ideally, when the participant really is a complex, then that complex itself should be identified with an external identifier like one from the Complex Portal at EBI (https://www.ebi.ac.uk/complexportal/home) [3]. In that case it is clear that all elements of such a complex are involved in the reaction, although the curator may still have made clear that one element is directly involved. In that case, the interaction will be graphically connected with an element inside the complex.

Also in the GitHub repository is a directory titled *pastReleases* with tables of values for the queries that were performed on the November 2016 release of the WikiPathways RDF as a comparison to the June 2019 release used in this paper. S4 File is also included as a zip file for the results of the June 2016 release. What is reflected in this comparison is that there is ongoing growth of the WikiPathways database and its semantic descriptions which sees a 43.8% increase in datanodes and an 23.3% increase in interactions from the 2016 release to the more recent release. All datanode types and interactions saw an increase in the later release compared to the earlier release, except for the case of stimulation interactions. This value went down between the releases as a result of curation efforts that identified that several of the interactions annotated as stimulations were incorrectly typed as such. Because of this curation the interactions were re-typed as their appropriate interaction type and thus we see a decrease in their number of interactions.

There is an ongoing discussion on user defined groups too, e.g. on how those should be connected and represented in the RDF as there might not be a single solution to address all the use cases of user groups. For example, these user groups often represent a class of enzymes that are all capable of catalyzing the same reaction, this can be seen in the example of the sphingolipid metabolism pathway, wikipathways:WP1423. Several intended interactions are not included in the WPRDF since the participants belong to a group of isoenzymes and will not be found in SPARQL query results. For this case, a simple solution would be to connect each element of the group via a duplicate interaction that is annotated as a catalysis towards the conversion, but not connect the isoenzymes to each other as is implied in the case of a biological complex. However, a user group could currently be any sort of convenient grouping and so this solution would not be a catch all solution for all groups, and further specifications would have to be included in the WikiPathways drawing options set itself.

The modelled biological knowledge of WikiPathways has previously been reported in the Waagmeester *et al*. paper [10]. During that analysis, the first release of WPRDF was explored to determine how elements were connected to one another in that semantic part of the RDF. As discussed above, there were many interactions that are drawn in the pathway and in the graphical information about a pathway but not found in the semantic layer. This was partly addressed by curation efforts that made sure that data nodes are drawn, typed and identified correctly and interactions are drawn for instance from anchors of the data nodes to another anchor in the drawing program. Overall 56% of interactions in the graphical information is now represented in the semantic portion. The WikiPathways connection information helps the WikiPathways team with their curation efforts with automated queries that have been implemented on the Jenkins platform [31].

Nevertheless, as was shown in the two biological examples above, it is possible to take advantage of the semantic information in the RDF to answer relevant questions. MECP2 was chosen as it is a signaling pathway and ganglio sphingolipid metabolism is a metabolic pathway. Both MECP2 and spingolipids are active research lines in the group. For MECP2, known to be a core epigenetic regulator, it was possible to identify MECP2 in pathway diagrams and then use connectivity information to find which other elements have a direct influence upon it and which elements MECP2 influences directly. In sphingolipid metabolism, conversion of metabolites from one form to another by a catalysis reaction were shown. This has interesting implications as it is then possible to expand this knowledge to infer information about the hierarchy of enzymes in this pathway. Meaning that, for example, GD3 is converted to GD2 by enzyme B4GALNT1 and GD2 is converted to GD1B by enzyme B3GALT4. This means that anything that acts upon and affects the activity of the upstream B4GALNT1 enzyme, will also affect the conversion of GD2 to GD1B by B3GALT4 through influence on substrate availability. This is more of an indirect influence of one element upon another but it is possible to then retrieve these indirect interactions.

The connectivity information from WikiPathways has already been deployed and taken advantage of in several instances. Pathway connectivity RDF information was integrated into the Open PHACTS Discovery Platform [32]. The connectivity information used in Open PHACTS was necessary to answer basic competency questions for the platform [33]. The connectivity information also became a useful way to create a network of pathways to identify active subnetworks in rare diseases [6]. This is part of a larger process involved with creating RDF of pathway data and using that information to answer questions in biology.

## Conclusion

It was demonstrated that most of the graphical biological knowledge from WikiPathways is modelled in the semantic layer (WPRDF) of WikiPathways RDF with the semantic information intact and connectivity information preserved. This semantic translation allows us to answer biological questions. The MECP2 example shows directional regulatory information captured by the WPRDF, and for the other example of sphingolipid metabolism complex successive biochemical reactions are captured. MECP2 involvement in regulatory, epigenetic interactions has implications for the understanding of the rare disease Rett syndrome. Sphingolipids are important parts of cell function and structure. Being able to evaluate the order in which biological elements affect each other allows, for example, the identification of up or downstream targets that will have a similar effect when modified.

The usability of the WikiPathways pathway and connectivity information has shown to be useful and has been integrated into platforms such as the Open PHACTS Drug Discovery Platform [32]. Improvements in WikiPathways curation and in the conversion to WikiPathways RDF support these other platforms and will allow giving a more complete picture of connectivity in biological systems. Continued curation efforts will incrementally improve many of the shortcomings of data and will continually make the semantic information better. The addition of shape expressions is a new method introduced that allows researchers to identify the form to expect from an interaction. Efforts to improve on the conversion scripts can address lost connectivity information that is for instance the result of using groups and complexes. Pathways themselves are also continually being added to WikiPathways and will continue to add to the richness of knowledge of biological interactions.

## Supporting information

S1 File NotFoundInWPRDF. Table for the top 20 datanodes that are found in the GPMLRDF but not in the WPRDF presented in the CSV file format accessible through most spreadsheet programs.
(CSV)

S2 File MECP2Stats. Table for the conversion statistics of the MECP2 pathway wikipathways:WP4312 in the CSV file format accessible through most spreadsheet programs.
(CSV)

S3 File SphingolipidStats. Table for the conversion statistics of the sphingolipid metabolism pathway wikipathways:WP1423 in the CSV file format accessible through most spreadsheet programs.
(CSV)

S4 File 201611RDFResults. Zip file for the tables of values for the queries that were performed on the November 2016 release of the WikiPathways RDF.
(ZIP)

S5 File ComplexRes. Table for the Complex GPMLRDF datanodes without WPRDF equivalents. This is the same 16 complexes identified in Table.
(CSV)

## Abbreviations

GPML: Graphical Pathway Markup Language
GPMLRDF: RDF for Graphical Pathway Markup Language
HGNC: HUGO Gene Nomenclature Committee
KEGG: Kyoto Encyclopedia of Genes and Genomes
MIM: Molecular Interaction Map
RDF: Resource Description Framework
SBGN: Systems Biology Graphical Notation
ShEx: Shape Expressions
SPARQL: SPARQL Protocol and RDF Query Language
WikiPathways RDF: The combination of GPMLRDF and WPRDF
WP: WikiPathways
WPRDF: RDF for WikiPathways
